# Two‐year outcomes of infants enrolled in the first‐in‐human study of amnion cells for bronchopulmonary dysplasia

**DOI:** 10.1002/sctm.19-0251

**Published:** 2019-11-27

**Authors:** Atul Malhotra, Rebecca Lim, Joanne C. Mockler, Euan M. Wallace

**Affiliations:** ^1^ Monash Newborn, Monash Children's Hospital Clayton Victoria Australia; ^2^ The Ritchie Centre Hudson Institute of Medical Research, Monash University Clayton Victoria Australia; ^3^ Department of Paediatrics Monash University Clayton Victoria Australia; ^4^ Department of Obstetrics and Gynaecology Monash University Clayton Victoria Australia

**Keywords:** long‐term, preterm, safety, stem cell, tumor

## Abstract

We previously reported on the immediate safety and neonatal outcomes of six premature infants with severe bronchopulmonary dysplasia (BPD) who were administered human amnion epithelial cells (hAECs). One infant died in the neonatal period due to unrelated causes. In this study, we aimed to assess the long‐term safety and follow‐up outcomes of the five surviving infants until 2 years corrected age (CA). hAECs were administered intravenously at a dose of 1 × 10^6^ cells per kilogram after 36 weeks postconceptional age in infants with established BPD. Study follow‐up consisted of assessment of any adverse events, growth, and respiratory, cardiac, and neurodevelopmental outcomes over four time points (6, 12, 18, and 24 months CA). Investigations included chest x‐rays, cranial and abdominal ultrasounds, and echocardiograms at regular intervals as well as a magnetic resonance imaging (MRI) brain at 2 years CA. All five infants were alive at 2 years CA. Median time to wean off oxygen was 24 (10‐36) months. Two infants had pulmonary hypertension, which resolved by 2 years of age. Four infants were rehospitalized briefly for viral or bacterial infections during the 2 years. MRI brain findings included normal (n = 1), and mild to moderate white matter loss (n = 2). Neurodisabilities diagnosed included hemiplegic cerebral palsy (n = 1), global developmental delay (n = 3), and severe hearing loss (n = 3). No evidence of tumor formation was noted on physical examinations or on any imaging. There were no long‐term adverse events observed that could be attributed to hAEC administration. We observed long‐term effects of extreme prematurity and severe BPD in the cohort.

## Graphical abstract

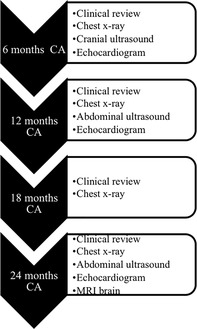

**Significance statement**

The long‐term safety results of this trial are an important addition to the literature to inform the scientific and clinical community about the impacts of this cell therapy.

## 1 INTRODUCTION

Despite advances in neonatal intensive care, bronchopulmonary dysplasia (BPD) continues to be a common complication in premature infants, especially those born extremely premature (<28 weeks' gestation).^1^, ^2^ BPD has significant impact on the health of the infant in the neonatal period and early childhood and leads to increased risk of long‐term respiratory issues, such as reactive airway disease, impaired lung function, and exercise intolerance.^3^, ^4^ Severe BPD is also associated with developmental delay and other neuromorbidities in children.^5^

At a pathophysiological level, BPD is characterized by vascular maldevelopment, impaired alveolarization, and lung inflammation.^6^ Stem cells have been reported to improve lung structure and function in preclinical animal models of BPD^7^, ^8^ and have been suggested as promising therapy to prevent and treat BPD.^9^, ^10^ Stem cell types used in preclinical models of BPD include mesenchymal stromal cells (MSCs), placental stem cells, and umbilical cord blood‐derived mononuclear cells.^10^, ^11^, ^12^ The amniotic membrane from the placenta is an abundant and reliable source of a particular type of stem cell, the human amnion epithelial cell (hAEC). Stem and stem‐like cells from the amniotic membrane are both immune privileged and immunomodulatory. This means that the risk of host rejection upon transplantation is low while the cells still retain potential disease‐modifying properties.^12^ The therapeutic utility of hAECs has been explored for a variety of disease conditions including lung disease, liver disease, and stroke in small and large animal models.^13^, ^14^, ^15^ In preclinical models of BPD, hAECs have been shown to prevent alveolar simplification and lung inflammation, two of the hallmarks of BPD,^7^ and to prevent pulmonary hypertension, a key sequelae of BPD.^15^

With a view to future clinical efficacy trials, we undertook a first‐in‐human phase I trial to assess the safety and tolerability of allogeneic hAECs in premature babies with established BPD.^16^ We have previously reported the immediate and short‐term outcomes until discharge from hospital.^16^ The aim of this further report was to describe the longer term safety outcomes of the infants until age 2, as originally detailed in the clinical trial protocol (ACTRN12614000174684).

## 2 MATERIALS AND METHODS

### 2.1 Ethics and consent

The study was approved by the Monash Health Human Research Ethics Committee (13324B). All human tissues for hAEC collection were obtained with written, informed patient consent, and a separate written, informed parental consent was obtained prior to administration of hAECs to the eligible infants.

### 2.2 hAEC collection, preparation, and release

The methods to collect, prepare, and release hAECs have been detailed previously.^16^ In brief, placentae from healthy women with an uncomplicated pregnancy undergoing an elective cesarean section at term gestation were collected at the time of delivery. The amnion was peeled from the underlying chorion, rinsed in sterile saline, soaked in antibiotic‐antimycotic solution, and then transferred to the cell isolation facility of Monash Health Translational Precinct's Cell Therapy and Regenerative Medicine Platform. There, hAECs were isolated and cryopreserved within a GMP‐grade Biospherix Xvivo system, as previously described.^17^ Cells were released for clinical use when the cell viability, as determined by trypan blue exclusion, was >80% at time of cryopreservation and when a cell isolate was proved free of microbial contamination after 14 days of culture. On the day of cell infusion, hAECs were retrieved from liquid nitrogen storage and washed in sterile saline prior to resuspension in saline at the desired concentration. For the first baby, hAECs were resuspended at a concentration of 2 × 10^6^ cells per milliliter saline. For subsequent babies, hAECs were resuspended at 0.325 × 10^6^ cells per milliliter saline to provide a post‐filter infusion concentration of 0.25 × 10^6^ cells per milliliter saline.

### 2.3 Infant selection and cell administration

Ex‐preterm infants (born ≤28^+0^ weeks' gestation) with established BPD at 36 weeks postconceptional age were eligible if they were dependent on invasive (mechanical ventilation) or noninvasive (continuous positive airway pressure [CPAP]) respiratory support in 0.3‐0.5 FiO_2_ at the time of cell administration. Infants with an active bacterial or viral infection, necrotizing enterocolitis, patent ductus arteriosus, or known severe brain injury at the time of administration were excluded. hAECs were given to three babies who were intubated before administering cells to a further three babies on CPAP support. All babies received the cells via a peripheral intravenous infusion. As previously described in detail,^16^ the first baby received the cell infusion by a slow, hand‐delivered injection. Subsequent babies received hAEC infusions delivered over 30 minutes by a syringe pump on a platform rocker. The dose of hAECs administered to all babies was 1 × 10^6^ cells per kilogram body weight at the time of delivery.

### 2.4 Outcomes and follow‐up protocol

**Figure 1:**
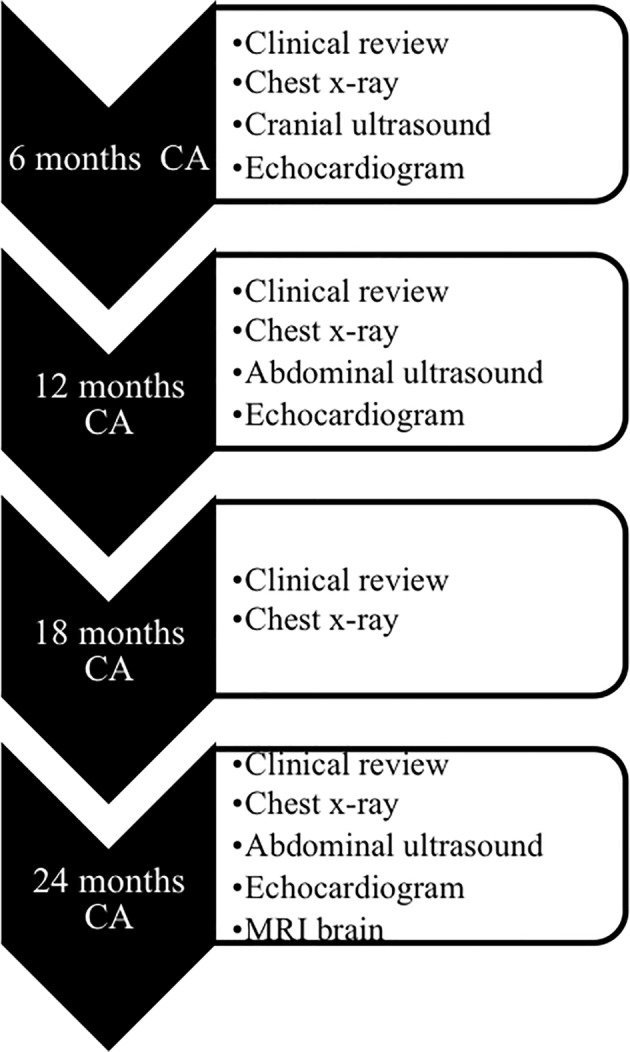
Schedule of follow‐up assessments

The primary outcome of this phase I trial was safety. The outcomes of the enrolled babies until discharge from hospital have been reported previously.^16^ In this paper, we report on the outcomes of the enrolled babies following discharge from the neonatal unit until 2 years of corrected age (CA). This included regular physical examinations, chest x‐rays, echocardiograms, and cranial and abdominal ultrasounds at set time points (6, 12, 18, and 24 months CA) as well as a magnetic resonance imaging (MRI) brain at 2 years CA, done according to a predefined trial protocol (ACTRN12614000174684). The schedule of the follow‐up clinical reviews and investigations at these time points is detailed in Figure 1. All infants also received routine medical and neurodevelopmental follow‐up as per unit policy for follow‐up of extremely premature and extremely low birth weight (ELBW) infants. Bayley Scales of Infant Development‐III was used for the neurodevelopmental assessment at 2 years CA, and given the severe nature of developmental delays in the infants, we have presented data as the developmental level they were determined to be at time of assessment in the three scales/domains: motor (gross and fine motor), language (expressive and receptive), and cognition.

## 3 RESULTS

### 3.1 Infant characteristics

Characteristics of the enrolled infants are summarized in Table 1. Six extremely preterm and ELBW infants (five boys, one girl) with a median (range) gestation at birth of 26 (24‐28) weeks and birth weight of 795 (450‐990) grams were administered hAECs at 89 (59‐187) postnatal days. One infant (baby 2) died a month after cell administration due to an unrelated cause.^16^ All five surviving infants were discharged home on supplemental low‐flow oxygen at a median of 174 (155‐388) days of life.

**Table 1:** Infant characteristics until discharge from NICU

Characteristics	Infant 1	Infant 2	Infant 3	Infant 4	Infant 5	Infant 6
Gestational age, weeks	27^+6^	28^+0^	25^+1^	25^+0^	27^+4^	24^+5^
Birth weight, grams	814	450	870	990	730	775
Sex	Male	Male	Male	Male	Male	Female
BPD, severity	Severe	Severe	Severe	Severe	Severe	Severe
Pulmonary hypertension	Yes	Yes	Yes	Yes	No	No
Postnatal age at hAEC, days	187	98	122	78	59	80
hAEC dose, million cells per kg	0.5	1	1	1	1	1
Death during NICU stay	No	Yes	No	No	No	No
Postnatal age at discharge, days	388	N/A	168	238	174	155
Home oxygen	Yes	N/A	Yes	Yes	Yes	Yes

Abbreviations: BPD, bronchopulmonary dysplasia; N/A, not applicable; NICU, neonatal intensive care unit.

### 3.2 Follow‐up outcomes

The follow‐up outcomes of the infants are detailed in Table 2. All infants were alive at 2 years of CA. Median time to wean off home oxygen was 24 (10‐36) months. Four infants were rehospitalized briefly for viral (three infants) and bacterial (one infant) infections during the first 2 years. Infant 3 presented to hospital three times during this time with an upper respiratory tract infection. There were no intensive care readmissions.

**Table 2:** Respiratory and other outcomes of study infants after NICU discharge until 2 years CA

Outcomes	Infant 1	Infant 3	Infant 4	Infant 5	Infant 6
Respiratory‐related hospitalizations	Nil	Yes, ×3 Viral	Yes, ×1 Croup	Yes, ×1 Bacterial bronchitis	Yes, ×1 Viral
ICU admissions	N/A	Nil	Nil	Nil	Nil
Age when in room air, months	33	24	20	9	12
Chest x‐ray abnormalities	Hyperinflated lungs (1 year) Interstitial thickening (2 years)	Hyperinflated lungs (1 year)	Chronic perihilar infiltrates (6 months to 2 years)	Hyperinflated lungs (2 years)	Nil
Reactive airway disease	Yes	No	No	Yes	Yes
Echocardiogram abnormalities	Trivial PDA, mild aortic root, ascending aortic dilatation, nonobstructive subaortic ridge	Small PDA	Small PDA	Nil	Nil
Cardiovascular diagnoses	Pulmonary hypertension	Nil	Pulmonary hypertension	Systemic hypertension (Normal renal scan)	Nil
Abdominal ultrasound abnormalities	Nil	Four lymph nodes in the right para‐aortic region with a maximum diameter of 7 mm. Both kidneys small (1 year) Lymph nodes of normal size and morphology (2 years)	Nil	Prominent portal tracts (1 year) Nil (2 years)	Nil
Cranial ultrasound abnormalities, 6 months	Mildly prominent ventricles	Nil	Nil	Mild bilateral prominent lateral ventricles	Bilateral ventricular size at the upper limits of normal Persistent prominent extra‐axial spaces
MRI brain abnormalities	Supratentorial mild white matter volume loss with thin corpus callosum and mild ventriculomegaly (2 years)	Incidental finding of small focus of susceptibility abnormality within the right cerebellar hemisphere (2 years)	Nil	Mild bilateral prominent lateral ventricles (2 years)	Punctate hyperintensity in the right centrum semiovale in the region of the corticospinal tracts (1 year)
Neurodevelopmental and neurosensory abnormalities	Hearing loss Developmental delay (9‐10 months level all domains) Myopia nystagmus Autism spectrum disorder	Hearing loss (aids) Developmental delay (15 months level motor, 4–5 months level cognition/language) Sleep disorder Behavioral disorder (under investigation)	Hearing loss (aids) Developmental delay (12‐18 months level motor/language, 24 months level cognition)	Hearing loss	Hearing loss (aids) Cerebral palsy (hemiplegic)
Physical examination abnormalities	Microcephaly Hypospadias (had operation)	Short stature	Nil	Nil Hypospadias (had operation)	Hemiparesis
Growth issues	Poor growth (PEG)	Nil	Nil	Poor growth (PEG)	Poor growth

*Note*: Infant 2 died during NICU stay, unrelated to trial participation.

Abbreviations: CA, corrected age; ICU, intensive care unit; MRI, magnetic resonance imaging; N/A, not applicable; NICU, neonatal intensive care unit; PDA, patent ductus arteriosus; PEG, percutaneous endoscopic gastrostomy.

There were no abnormalities on physical examination noted that were suggestive of tumor formation. Failure to thrive and poor growth was an issue in three infants. This required agastrostomy insertion in two children. Weight‐related growth issues resolved by 2 years CA in all infants.

Serial (every 6 months until 2 years CA) chest x‐rays in the infants showed variable changes (hyperinflated lung fields, interstitial thickening, perihilar infiltrates) related to BPD sequelae. Three of the five infants were diagnosed with reactive airway disease by 2 years CA. All of them were on reliever (inhaled beta 2 agonist) medications for their symptoms, and one of them was also on a preventer (inhaled steroids).

Two infants had pulmonary hypertension at the time of discharge and received oral sildenafil until resolution. In both children, this had resolved by 2 years of age. One infant had associated systemic hypertension (received one antihypertensive—captopril), which also resolved by 18 months of age. There were no renal or cardiac abnormalities noted. Echocardiograms revealed trivial or small patent ductus arteriosus in three infants. One infant also had mild aortic root and ascending aortic dilatation with a nonobstructive subaortic ridge. There were no other abnormalities noted in cardiac structure or function.

Abdominal ultrasounds were done at 12 and 24 months of CA. In infant 3, multiple para‐aortic lymph nodes, reported as reactive, were seen at the 12  months CA scan. The scan was repeated after 6 months, confirming resolution of the lymph nodes. In infant 5, prominent portal tracts were seen at the 12 months CA scan. These were normal on a repeat scan 6 months later.

Cranial ultrasound was repeated once after discharge at 6 months of CA. Mildly prominent ventricles were seen in three of the five infants. MRI brain findings included normal (n = 1), mild white matter loss with prominent ventricles (n = 2), incidental susceptibility abnormality in the cerebellum (n = 1), and punctate hyperintensity in centrum semiovale (n = 1). Neurodisabilities in the infants included hemiplegic cerebral palsy (n = 1) and global developmental delay (n = 3). The developmental delay was severe in all three infants, precluding exact scores for testing scales to be assigned; instead, developmental levels at time of assessment were assigned for each scale (cognitive, language, and motor), as detailed in Table 2. Behavioral issues (one with autism spectrum disorder and another one under investigation) were present in two infants. Hearing loss was diagnosed in all five infants, with three requiring hearing aids. One infant was diagnosed with a vision abnormality (myopia). All infants were ambulant and independently feeding at 2 years CA.

## 4 DISCUSSION

This is the report of the 2‐year outcomes of infants enrolled in the first‐in‐human trial of hAECs for BPD. All five surviving infants were alive at 2 years CA and there were no hAEC‐related complications. The infants had a spectrum of morbidities, all of which could be attributed to extreme prematurity and severe BPD. These observations provided further reassurance to assessment of potential efficacy of hAECs as a treatment for BPD and other neonatal conditions.

There were a number of complications seen in the study infants, which included growth problems, readmissions to the hospital for infections, and neurodevelopmental and neurosensory morbidities in the 2 years of follow‐up. These complications are unfortunately very common in ELBW infants with severe or significant BPD.^18^, ^19^ The study participants were infants with severe BPD who spent long periods of their initial life in hospital because of respiratory morbidity. Prolonged hospitalization is known to be a marker for poor outcome in the preterm population.^20^, ^21^ As the primary outcomes of this trial were safety related and there was no control group, it is not possible for us to conclude on any long‐term impact of hAEC treatment on these clinical morbidities. Such insights will only be afforded by future randomized controlled trials. Nonetheless, we did not observe any outcomes that we could attribute to hAECs, and it is unlikely that the low dose of hAEC administration had any consequence on their long‐term health. It was also not surprising that the complications occurred in these five infants. All of the infants had established BPD at the time of cell administration. It was not expected that hAEC administration would have any disease‐modifying effects in this first‐phase trial.

Apart from this study, there are two other published cell therapy trials for BPD.^22^, ^23^ One was an open‐label, single‐center trial of proprietary human umbilical cord blood‐derived MSCs, Pneumostem.^22^ In the trial, nine premature infants were administered MSCs between 1 and 2 weeks of life at doses of 1 × 10^7^ cells per kilogram or 2 × 10^7^ cells per kilogram through the intratracheal route.^22^ No serious acute adverse effects associated with MSC delivery were reported, and infants were followed up for 2 years with no adverse outcomes in growth, respiratory, or neurodevelopmental function compared with a historical control group.^24^ More recently, Powell et al published their experience of intratracheal administration of a single dose (also at 1 × 10^7^ or 2 × 10^7^ cells per kilogram of human umbilical cord blood‐derived MSCs (Pneumostem) in 12 ELBW infants at 5‐14 days of life.^23^ Again, the cell administration was well tolerated. Long‐term follow‐up for the study infants is ongoing. A phase II randomized, double‐blind, multicenter trial of 70 babies to further assess safety and efficacy of intratracheal MSCs (Pneumostem) has recently been completed but not yet reported (NCT01828957). In older children and adults, a meta‐analysis of MSC treatment to more than 1,000 participants for a variety of clinical indications also found no evidence of adverse events, including surveillance for malignancy up to 5 years following cell delivery.^25^ These trials are providing cumulative reassurance to parents and clinicians that cell therapy for BPD appears safe, at least to 2 years of age.

It is likely that much higher doses of cells will be required for efficacy trials.^26^ With that in mind, we have commenced a multicenter dose escalation study of hAECs given to extremely premature infants at high risk of developing BPD in the second to third week of life. This study is also primarily a safety trial, designed to assess whether the total cell doses that will likely be necessary for any therapeutic effect, 20‐30 million cells per kilogram, are tolerated by extremely preterm neonates within the first weeks of life.^26^ The doses projected in the dose escalation study are up to 30 times the dose given in the current study. This is based on preclinical studies on lung disease models using hAECs and is also in line with clinical trials using umbilical cord blood stem cells for brain injury,^27^ cerebral palsy,^28^ and autism.^29^ The results of that dose escalation trial will be critical for designing the next phase of our translational work in hAECs for the prevention and treatment of BPD.

## 5 CONCLUSION

The long‐term follow‐up outcomes of our first‐in‐human study of hAECs for BPD are reassuring and indicate that low‐dose allogeneic hAECs are safe to administer to vulnerable premature infants. Ongoing studies will help elucidate the optimum dosage and timing of cell administration to high‐risk preterm infants before we embark on conclusive efficacy trials of hAECs for prevention of BPD.

## CONFLICT OF INTEREST

R.L. declared employment/leadership position with Meluha Capital, inventor or patent holder Regenasome Pty Ltd, honoraria received from Thermo Fisher Scientific, and research funding from United Therapeutics. The other authors indicated no potential conflicts of interest.

## AUTHOR CONTRIBUTIONS

A.M.: concept and design, recruitment, data collection and analysis, manuscript writing, and final approval; R.L.: concept and design; cell preparation; manuscript editing, and final approval; J.C.M.: manuscript editing, follow‐up coordination, and final approval; E.M.W.: concept and design, manuscript editing, financial support, and final approval.

## Data Availability

The data that support the findings of this study are available on request from the corresponding author. The data are not publicly available due to privacy or ethical restrictions.

## References

[1] Jobe AH , Bancalari E . Bronchopulmonary dysplasia. Am J Respir Crit Care Med. 2001;163:1723‐1729. 11401896 10.1164/ajrccm.163.7.2011060

[2] Higgins RD , Jobe AH , Koso‐Thomas M , et al. Bronchopulmonary dysplasia: executive summary of a workshop. J Pediatr. 2018;197:300‐308. 29551318 10.1016/j.jpeds.2018.01.043 PMC5970962

[3] Doyle LW , Carse E , Adams AM , et al. Ventilation in extremely preterm infants and respiratory function at 8 years. N Engl J Med. 2017;377:329‐337. 28745986 10.1056/NEJMoa1700827

[4] Doyle LW , Ranganathan S , Cheong JLY . Ventilation in preterm infants and lung function at 8 years. N Engl J Med. 2017;377:1601‐1602. 29045215 10.1056/NEJMc1711170

[5] Cheong JLY , Doyle LW . An update on pulmonary and neurodevelopmental outcomes of bronchopulmonary dysplasia. Semin Perinatol. 2018;42:478‐484. 30401478 10.1053/j.semperi.2018.09.013

[6] Baker CD , Alvira CM . Disrupted lung development and bronchopulmonary dysplasia: opportunities for lung repair and regeneration. Curr Opin Pediatr. 2014;26:306‐314. 24739494 10.1097/MOP.0000000000000095 PMC4121955

[7] Vosdoganes P , Hodges RJ , Lim R , et al. Human amnion epithelial cells as a treatment for inflammation‐induced fetal lung injury in sheep. Am J Obstet Gynecol. 2011;205:156.e126‐e133. 10.1016/j.ajog.2011.03.054 21640967

[8] Hodges RJ , Jenkin G , Hooper SB , et al. Human amnion epithelial cells reduce ventilation‐induced preterm lung injury in fetal sheep. Am J Obstet Gynecol. 2012;206:448.e448‐e415. 10.1016/j.ajog.2012.02.038 22542124

[9] Nitkin CR , Rajasingh J , Pisano C , et al. Stem cell therapy for preventing neonatal diseases in the 21st century: current understanding and challenges. Pediatr Res. 2019; [Epub ahead of print]. 10.1038/s41390-019-0425-5 PMC6854309 31086355

[10] Vosdoganes P , Lim R , Moss TJ , et al. Cell therapy: a novel treatment approach for bronchopulmonary dysplasia. Pediatrics. 2012;130:727‐737. 22945412 10.1542/peds.2011-2576

[11] O'Reilly M , Thebaud B . Cell‐based therapies for neonatal lung disease. Cell Tissue Res. 2017;367:737‐745. 27770256 10.1007/s00441-016-2517-4

[12] Zhu D , Wallace EM , Lim R . Cell‐based therapies for the preterm infant. Cytotherapy. 2014;16:1614‐1628. 25154811 10.1016/j.jcyt.2014.06.004

[13] Evans MA , Lim R , Kim HA , et al. Acute or delayed systemic administration of human amnion epithelial cells improves outcomes in experimental stroke. Stroke. 2018;49:700‐709. 29382802 10.1161/STROKEAHA.117.019136

[14] Tan JL , Lau SN , Leaw B , et al. Amnion epithelial cell‐derived exosomes restrict lung injury and enhance endogenous lung repair. Stem Cells Translational Medicine. 2018;7:180‐196. 29297621 10.1002/sctm.17-0185 PMC5788876

[15] Zhu D , Tan J , Maleken AS , et al. Human amnion cells reverse acute and chronic pulmonary damage in experimental neonatal lung injury. Stem Cell Res Ther. 2017;8:257. 29126435 10.1186/s13287-017-0689-9 PMC5681809

[16] Lim R , Malhotra A , Tan J , et al. First‐in‐human administration of allogeneic amnion cells in premature infants with bronchopulmonary dysplasia: a safety study. Stem Cells Translational Medicine. 2018;7:628‐635. 30078207 10.1002/sctm.18-0079 PMC6127230

[17] Murphy S , Rosli S , Acharya R , et al. Amnion epithelial cell isolation and characterization for clinical use. *Curr Protoc Stem Cell Biol* 2010;Chapter 1:Unit 1E.6. 10.1002/9780470151808.sc01e06s13 20373516

[18] Kuint J , Lerner‐Geva L , Chodick G , et al. Rehospitalization through childhood and adolescence: association with neonatal morbidities in infants of very low birth weight. J Pediatr. 2017;188:135‐141.e132. 28662947 10.1016/j.jpeds.2017.05.078

[19] Islam JY , Keller RL , Aschner JL , Hartert TV , Moore PE . Understanding the short‐ and long‐term respiratory outcomes of prematurity and bronchopulmonary dysplasia. Am J Respir Crit Care Med. 2015;192:134‐156. 26038806 10.1164/rccm.201412-2142PP PMC4532824

[20] Choi YB , Lee J , Park J , Jun YH . Impact of prolonged mechanical ventilation in very low birth weight infants: results from a national cohort study. J Pediatr. 2018;194:34‐39.e33. 29198532 10.1016/j.jpeds.2017.10.042

[21] Rodriguez‐Martinez CE , Acuna‐Cordero R , Sossa‐Briceno MP . Predictors of prolonged length of hospital stay or readmissions for acute viral lower respiratory tract infections among infants with a history of bronchopulmonary dysplasia. J Med Virol. 2018;90:405‐411. 28975628 10.1002/jmv.24962

[22] Chang YS , Ahn SY , Yoo HS , et al. Mesenchymal stem cells for bronchopulmonary dysplasia: phase 1 dose‐escalation clinical trial. J Pediatr. 2014;164:966‐972.e966. 24508444 10.1016/j.jpeds.2013.12.011

[23] Powell SB , Silvestri JM . Safety of intratracheal administration of human umbilical cord blood derived mesenchymal stromal cells in extremely low birth weight preterm infants. J Pediatr. 2019;210:209‐213.e202. 30992220 10.1016/j.jpeds.2019.02.029

[24] Ahn SY , Chang YS , Kim JH , et al. Two‐year follow‐up outcomes of premature infants enrolled in the phase I trial of mesenchymal stem cells transplantation for bronchopulmonary dysplasia. J Pediatr. 2017;185:49‐54.e42. 28341525 10.1016/j.jpeds.2017.02.061

[25] Lalu MM , McIntyre L , Pugliese C , et al. Safety of cell therapy with mesenchymal stromal cells (SafeCell): a systematic review and meta‐analysis of clinical trials. PLoS One. 2012;7:e47559. 23133515 10.1371/journal.pone.0047559 PMC3485008

[26] Baker EK , Malhotra A , Lim R , Jacobs SE , Hooper SB , Davis PG , Wallace EM Human amnion cells for the prevention of bronchopulmonary dysplasia: a protocol for a phase I dose escalation study. BMJ Open 2019;9:e026265. 10.1136/bmjopen-2018-026265 PMC6398764 30826799

[27] Cotten CM , Murtha AP , Goldberg RN , et al. Feasibility of autologous cord blood cells for infants with hypoxic‐ischemic encephalopathy. J Pediatr. 2014;164:973‐979.e971. 24388332 10.1016/j.jpeds.2013.11.036 PMC3992180

[28] Sun JM , Song AW , Case LE , et al. Effect of autologous cord blood infusion on motor function and brain connectivity in young children with cerebral palsy: a randomized, placebo‐controlled trial. Stem Cells Translational Medicine. 2017;6:2071‐2078. 29080265 10.1002/sctm.17-0102 PMC5702515

[29] Dawson G , Sun JM , Davlantis KS , et al. Autologous cord blood infusions are safe and feasible in young children with autism spectrum disorder: results of a single‐center phase I open‐label trial. Stem Cells Translational Medicine. 2017;6:1332‐1339. 28378499 10.1002/sctm.16-0474 PMC5442708

